# mRNA Vaccines for COVID-19: A Simple Explanation

**DOI:** 10.5339/qmj.2021.7

**Published:** 2021-02-18

**Authors:** Mohamed A. Hendaus, Fatima A. Jomha

**Affiliations:** ^1^Department of Pediatrics, Sidra Medicine, Doha, Qatar E-mail: mhendaus@yahoo.com; ^2^Weill Cornell Medicine Qatar, Doha, Qatar; ^3^School of Pharmacy, Lebanese International University

## Introduction

As with the introduction of any novice agent, the coronavirus-19 (COVID-19) vaccine is causing fear and concern about its safety and efficacy in certain populations. This fear could be attributed to the inadequate public knowledge of this vaccine. The currently introduced COVID-19 vaccines are based on messenger ribonucleic acids (mRNA). This commentary will provide a brief background about COVID-19 mRNA vaccine, its mechanism of action, and its safety, including its testing phases.

### The immune system

The immune system is composed of innate and adaptive components. The innate immunity is present at birth and comprises cells such as natural killer cells and neutrophils, which attack foreign cells in the body.^1^ On the other hand, the adaptive system comprises lymphocytes (type of white blood cell), either T or B cells, and includes both CD8+ killer and CD4+ helper T cells that regulate CD8+ T cells.^2^

In addition to the T cells, the adaptive immune system comprises B cells that produce antibodies against invaders. Antibodies are good at stopping a virus outside the cell. Once a virus infects the cell, then T cells pick up the battle. The immune system memory works similar to human's memory. Once it encounters an outsider, it will recognize or remember it in the future.^3^

### COVID-19 structure

**Figure 1.**
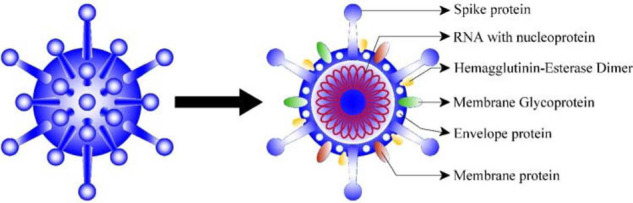
Molecular structure of coronavirus.

The coronavirus family has a sizable homogeneous ‘spike (S) protein’ (Figure. 1). The role of the S protein, which is composed of 1300 amino acids (units that make up a protein), is to interact with host cells and assist the coronavirus, making its way through the epithelial cell membrane.^4^,^5^

### mRNA vaccine versus conventional vaccines?

Most conventional vaccines use either live or attenuated (weakened) pathogens to boost the body's immune response. Live ‘bugs’ are usually found in the oral polio vaccine and in subcutaneous vaccines such as measles, mumps, rubella, and varicella. These conventional vaccines take a long time (years to decades) to manufacture, test, and get approved. In addition, they are expensive to produce.^5^

The white blood cells, which are considered the body's defense soldiers, sense a pathogen and frame a defense against it by generating specific antibodies to combat it.^3^

COVID-19 vaccine targets the S protein (Figure 1) via mRNAs, the molecule that instructs cells what to build.^6^ Once the S protein is produced within the body, it is considered an antigen, and the body starts producing antibodies to fight the real disease, if contracted.^7^

This type of vaccine was chosen mainly because it can be manufactured quickly, although several testing phases are being implemented. Furthermore, its production is laboratory-based, and the process is standardized. Besides vaccines, mRNA technology can also be used to develop and boost the immune system to fight precise cancer cells in the oncology field.^6^

The fastest way to make a vaccine in the midst of a pandemic is allowing the body to mount the production of antibodies against a protein on the surface of a virus (S protein in the case of COVID-19). These antibodies cover the virus and prevent it from attacking the body. Antibodies alone do not suffice for proper protection, so a special type of white blood cell (T cell) is needed.^7^ This process typically takes a few weeks for the body to produce the antibodies.^8^

**Figure 2.**
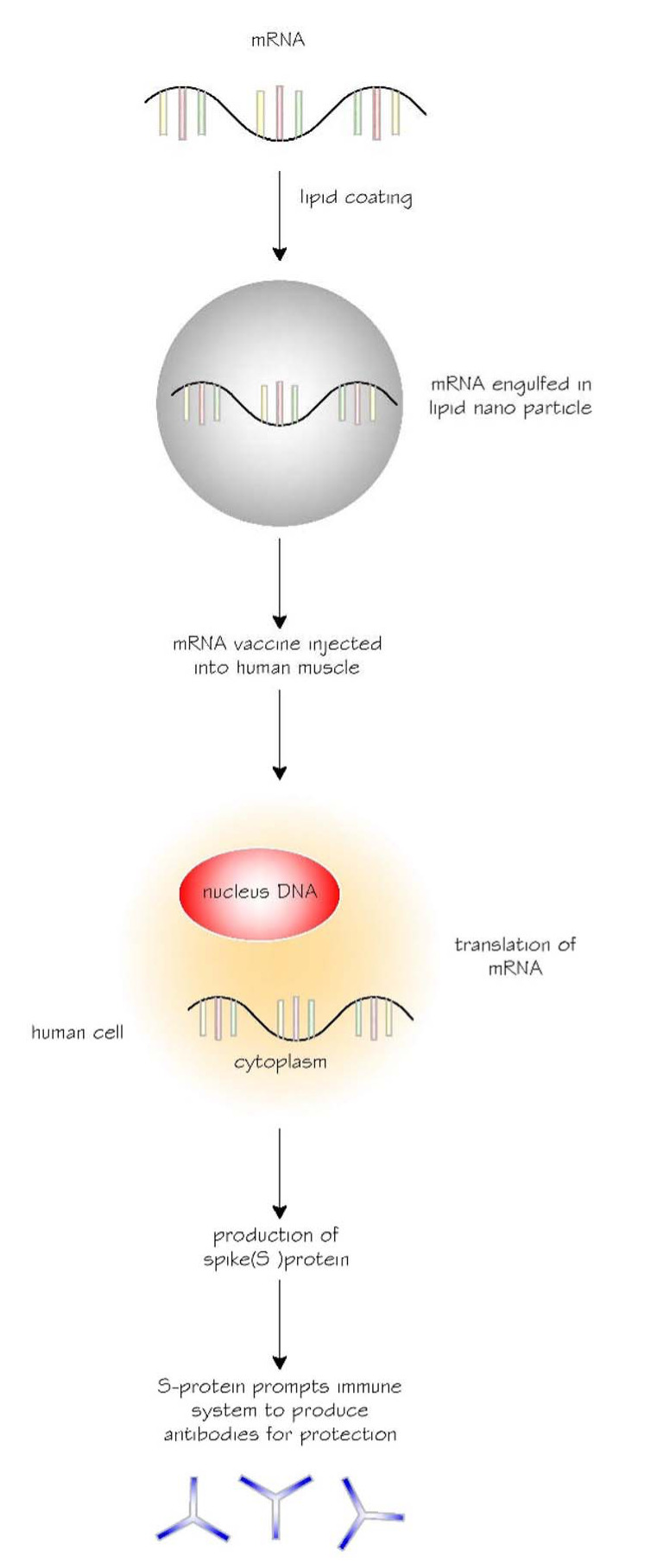
The journey of messenger ribonucleic acid (mRNA) vaccine in the human body. S protein, spike protein.

RNA is a common molecule in the body, and all living things use thousands of RNAs as messages to encode within the cells. mRNAs are meant to be transient or function for a short period (minutes to hours).^6^ An analogy to mRNAs is a ‘post-it note.’^9^ Once the post-it note is used, it will be shredded or discarded. An mRNA vaccine operates the same way. Once the mRNA portion of the vaccine is injected into the body (Figure 2), it travels to the cell through lipid (fat) and nano (small) particles.^6^ The lipid carrier is degraded once it reaches the cytoplasmic part of the cell, exposing the mRNA and instructing the immune system. The specific instruction is to create an immune response to the S protein by recognizing it as foreign and building an immune response.^10^ In the cytoplasm, the cascade of protein synthesis is triggered in the cellular organelles known as the ribosomes. Once the message is read, an mRNA eventually disintegrates. Therefore, mRNAs are merely temporary messages.^9^ It is important to note that mRNA functions in the cytoplasm of the cell and does not interact with the nucleus. Therefore, it does not affect, modify, or mutate the genetic material, deoxyribonucleic acid, of the body.^10^

Another option to boost the immune system against the S protein is vaccinating individuals with the protein itself, similar to the case of the diphtheria tetanus acellular pertussis.^11^ However, the process of manufacturing a protein in the laboratory is complex, lengthy, and expensive.

## Vaccine Testing Process

Clinical investigators and institutional review boards/independent ethics committees are always involved in determining the protection of a participant's safety, rights, and welfare.^12^

*Preclinical testing:* New vaccines are tested on animals, such as monkeys and mice, and are being checked for proper immune response. An mRNA vaccine has successfully passed the preclinical testing phase.^13-15^

*Phase 1 safety trials:* New vaccines are tested on a small number of human beings (usually between 20 and 80 subjects)^16^ and are being checked for safety and proper immune response.^14,15^ An mRNA vaccine has successfully passed the first phase of safety trial.^13-15^

*Phase 2 expanded trials:* New vaccines are tested on several hundred^16^ individuals and are being checked for safety and proper immune response. An mRNA vaccine has successfully passed the second phase of expanded trial.^13-15^

*Phase 3 efficacy trials:* New vaccines are tested on thousands to tens of thousands^16^ of human beings and are being checked for safety and proper immune response.^12,13^ An mRNA vaccine has successfully passed the third phase of efficacy trial that has been used on thousands of volunteers. Moreover, phase 3 trials are large enough to divulge data of relatively rare side effects that might be overlooked in earlier studies.^13-15^

*Approval*: Local officials in each country usually analyze the results of investigators and decide whether to introduce the vaccine or not. Vaccines sometimes receive emergency use authorization in the midst of a pandemic.^17^ Data monitoring will ensue to assure safety and effectiveness of the vaccine.^13-15^

*Paused*: If the data monitoring safety board observes perturbing symptoms in volunteers, a trial can be put on pause. The trial might be resumed or suspended, depending on the investigation outcome.^13-15^

### Effectiveness of mRNA vaccines against COVID-19

The effectiveness of Pfizer and Moderna vaccines ranges from 90% to 95%.^13,18,19^ The administration of two doses of the vaccines is common. The first dose exposes the human immune system to the S protein (antigen), and the second one boosts the memory cells. The same concept applies to different immunization schedule such as the influenza vaccine.^20^

It is unclear how long does the protective immunity last after a person contracted the COVID-19 viral infection. Therefore, the vaccine is recommended even for those who have had the disease. The literature has shown that vaccines might boost better immunity than the disease itself as seen in with the human papilloma virus vaccine.^21^

### Side effects and safety of mRNA COVID-19 vaccines

Thousands of individuals will have received the vaccine as part of the third phase of clinical trials, and data-monitoring review boards will have determined if the vaccine is safe.^13-15^

It is crucial to mention that the vaccine process was expedited in terms of the production phase. However, there were no shortcuts in terms of assessing their safety.^22^

Fatigue, fever, soreness, and local injection site reactions are common symptoms after vaccination.^23^ These symptoms are part of the anticipated response to the vaccine and imply that vaccine is working and prompting a robust immune response. Some scientists consider these symptoms as expected immunogenic and not ‘side effects.’^24^

All COVID-19 vaccine companies have reported low percentage of unpleasant reactions to the vaccine. For example, of 1,893,360 first doses Pfizer-BioTech COVID-19 vaccines administered, only 4393 have reported unpleasant reaction (0.2%).^25^

## Conclusions

COVID-19 mRNA vaccines are relatively safe and effective. The future of mRNA vaccines for other diseases is bright. This technology can eventually be used to lessen the need for administering multiple injections.
